# Difference in cerebral saturation during cardiopulmonary resuscitation between survivors with favorable neurological outcome and compromised neurological outcome at hospital discharge

**DOI:** 10.1186/cc14509

**Published:** 2015-03-16

**Authors:** C Genbrugge, W Boer, I Meex, F Jans, C Deyne, J Dens

**Affiliations:** 1 ZOL, Genk, Belgium; 2 Hasselt University, Hasselt, Belgium

## Introduction

During out-of-hospital cardiac arrest (OHCA) monitoring possibilities are limited. Recently, the role of cerebral oximetry, using near-infrared spectroscopy, during ALS was investigated. In this study we determined whether the magnitude of increase in cerebral saturation (rSO_2_) or mean rSO_2_ during prehospital ALS was associated with good neurological outcome at hospital discharge (Cerebral Performance Category (CPC) 1 or 2).

## Methods

With IRB approval, we prospectively measured rSO_2_ during ALS in consecutive OHCA patients. One sensor of the Equanox™ 7600 (NONIN) was applied on the patient's forehead's right side when the medical emergency team arrived in an OHCA. ROSC was defined as ROSC >20 minutes.

## Results

We included 88 prehospital cardiac arrest patients between December 2011 and October 2014 with eight (9%) patients with CPC 1 or 2. Twenty-seven patients of the nonsurvivors had ROSC >20 minutes and one patient had CPC 3 at hospital discharge. We observed no significant difference between both groups in age (*P *= 0.161), time between emergency call and start of ALS (*P *= 0.788) and duration of basic life support performed by bystanders, general practitioners or paramedics (*P *= 0.649). The initial rhythm was asystole in one (12.5%) survivor and in 50 (62.5%) nonsurvivors (*P *= 0.009), ventricular fibrillation in six (75%) survivors and 13 (16%) nonsurvivors (*P *= 0.001), and pulseless electrical activity in one (12.5%) survivor and 17 (21%) nonsurvivors (*P *= 1.00). The cardiac arrest was witnessed in all survivors (100%) and in 49 (61%) nonsurvivors (*P *= 0.046). First measured rSO_2 _was 38% (27 to 67) in the survivor group compared with 22% (8 to 32) in the nonsurvivor group (*P *= 0.004). Also a significant difference was found in mean rSO_2_ until 1 minute before ROSC between survivors and nonsurvivors, respectively 46% (40 to 74) and 34% (22 to 42). We observed no significant difference in increase of rSO_2_ until 1 minute before ROSC between survivors 12.5% (5 to 21) and nonsurvivors 11% (5 to 18) (*P *= 0.719).

## Conclusion

We observed a significant difference in first measured rSO_2 _and mean rSO_2_ until 1 minute before ROSC between patients with good neurological outcome (CPC 1 or 2) at hospital discharge and patients with worse neurological outcome or nonsurvivors (CPC 3 or 4 or 5). However, no significant difference was observed in the increase between both groups. Larger studies are necessary to confirm these results.

